# Heart rate variability in patients being treated for dengue viral infection: new insights from mathematical correction of heart rate

**DOI:** 10.3389/fphys.2014.00046

**Published:** 2014-02-25

**Authors:** Robert Carter, Carmen Hinojosa-Laborde, Victor A. Convertino

**Affiliations:** U.S. Army Institute of Surgical ResearchFort Sam Houston, TX, USA

**Keywords:** dengue hemorrhagic fever, heart rate variability, high power frequency, parasympathetic nervous system, autonomic balance

## Abstract

**Introduction:** Severe dengue hemorrhagic fever (DHF) is a viral infection that acts to increase permeability of capillaries, resulting in internal hemorrhage. Linear frequency domain Fourier spectral analysis represents the most published noninvasive tool for diagnosing and assessing health status via calculated heart rate variability (HRV). As such, HRV may be useful in assessing clinical status in DHF patients, but is prone to erroneous results and conclusions due to the influence of the average HR during the time period of HRV assessment (defined as the “prevailing” HR). We tested the hypothesis that alterations in HRV calculated with linear frequency analysis would be minimal when mathematically corrected for prevailing HR following dengue viral infection.

**Methods:** Male (*N* = 16) and female (*N* = 11) patients between the ages of 6 months and 15 years of age (10 ± 6 SD years) were tracked through the progression of the dengue viral infection with treatment following the abatement of a fever (defervescence). Electrocardiographic recordings were collected and analyzed for HRV.

**Results:** High frequency (HF), low frequency (LF), and LF/HF ratio were unaffected by correction for prevailing HR.

**Conclusion:** HRV corrected for changes in HR did not alter the interpretation of our data. Therefore, we conclude that cardiac parasympathetic activity (based on HF frequency) is responsible for the majority of the HR reduction following defervescence in patients with dengue viral infection.

## Introduction

Heart rate variability (HRV) represents a noninvasive “vital sign” that can be easily calculated in real-time from the R-to-R interval of the electrocardiogram (ECG). Low HRV has been recognized as reflecting more severe pathophysiology as reported in ICU patients (Winchell and Hoyt, 1996; Grogan et al., 2005; Morris et al., 2006; Norris et al., 2008; Ryan et al., 2008), experimental human models of hemorrhage (Convertino et al., 2008; Cooke et al., 2008; Ryan et al., 2010), and following injury in trauma (Cooke et al., 2006a,b; Cancio et al., 2008; Ong et al., 2008; King et al., 2009), while high HRV has been used as an indication of improved health. However, these reported results were based on linear analyses of HR calculated in the time and frequency domains. Importantly, linear frequency analysis of HRV is significantly affected by both physiological and mathematical factors as a result of the nonlinear relationship between R -R intervals and HR (Sacha and Pluta, 2005). As such, the use of HRV metrics to provide an accurate assessment of the effectiveness in the treatment of hemorrhage relies on the assumption that the mathematical influence of is the average HR during the time period of HRV assessment [defined as the “prevailing” HR] on HRV is not present or has been corrected. This assumption is reasonable when HR is not different between clinical populations of comparison or over time in the same patient population. But in the absence of mathematical adjustment of differences in HR by dividing R-R intervals by the corresponding average R-R, accurate interpretation of changes in HRV may be severely compromised (Sacha and Pluta, 2005, 2008; Billman, 2011, 2013b; Sacha, 2013; Sacha et al., 2013a,b).

Severe dengue hemorrhagic fever (DHF) is a viral infection that acts to increase permeability of capillaries, resulting in internal hemorrhage. The diagnosis and management of dengue vascular permeability syndrome has been one of the greatest challenges over the past fifty plus years since dengue shock syndrome was first described (Cohen and Halstead, 1966; Halstead et al., 1970). Since HR decreases from the time of patient hospital admission to the time of discharge (Yacoub et al., 2012), we considered the potential use of HRV with confounding changes in HR as an opportunity to determine the usefulness of HRV to assess the effectiveness of in-hospital treatment and to provide clearer insight into the physiology underlying the association of changes in HR with the recovery from DHF.

In the present investigation, we had the unique opportunity to monitor R-to-R interval measurements from patients with dengue viral infection during their hospitalization in order to capture HRV during the progression of the disease and treatment following the day of the abatement of a fever (defervescence). This approach provided the opportunity to assess the mathematical influence of HR on HRV in a patient population with internal hemorrhage by comparing linear measures of HRV with and without mathematical correction as a potential indicator of the effectiveness of treatment. Although Sacha and co-workers (Sacha and Pluta, 2005, 2008; Sacha et al., 2013b) have recently examined the relationship between average HR and indices of HRV under baseline conditions and compared methods to correct HRV for HR, the effects of HR on HRV during dengue fever (DF) disease progression and treatment remained to be determined. We hypothesized that alterations in HRV calculated with linear frequency analysis would be minimal or eliminated when mathematically corrected for changing HR under these unique conditions.

## Materials and methods

Male (*N* = 16) and (*N* = 11) female patients between the ages of 6 months and 15 years of age (10 ± 6 SD years) who were admitted to the Queen Sirikit National Institute of Child Health (QSNICH) with fever and suspected dengue were eligible for enrollment. Exclusion criteria for the study included known chronic conditions (e.g., liver and renal disease, malignancy, thalassemia). Informed consent from a parent or guardian was provided for all study procedures. The study was approved by the hospital Institutional Review Board, the Thai Ministry of Public Health, the US Army Surgeon General, and the University of Massachusetts Medical School. In order to track the progression of the dengue viral infection with treatment following defervescence, we used data collected on days 0 (defervescence), 1, and 2. Day 0 ranged from 0 to 3 days (mean 1 ± 0.9 SD days) following admission to the hospital. All data used in this study were collected in the morning (07.00–10.00) while patients were in the supine position (i.e., hospital bed).

Electrocardiographic recordings were collected in using a Nexfin (BMEye, Amsterdam, the Netherlands) at a sampling rate of 1000 Hz and exported at a rate of 200 Hz to a computer-based data acquisition software package (WinDAQ, Dataq Instruments, Akron, OH). The ECG waveforms were imported into data analysis software (WinCPRS, Absolute Aliens, Turku, Finland) using a Labview application for automatic R-wave detection. Due to the 200-Hz sampling rate, a smoothing filter of a 5-point running average was applied to the ECG data to provide clear peaks for R-wave generation. This filter application produced 0.5–1.0 s of data to be cut from each of the ECG waveforms. All signals were manually scanned for noise and missing R-wave detection. ECG recordings were discarded if they contained less than five minutes of data, more than one ectopic beat during any 5-min time span, or contained electromechanical noise or interference. Aberrant beats in the ECG recording were interpolated, most occurring from calibration or patient movement.

HRV measurements were assessed with analysis of R-R intervals (the time between the two successive R waves in ECG) using frequency domain methods obtained from 300-s continuous recordings with the least amount of aberrant beats. Using WinCPRS software, the following metrics were obtained according to a previously described approach (Ryan et al., 2010) RRI, heart rate (HR), RRI low frequency power (LF), RRI high frequency power (HF), and LF/HF ratio. However, HRV measurements have been shown to be significantly associated with HR due to both physiological and mathematical reasons. In order to remove mathematical bias from our HRV calculations, we used the HR correction methodology previously described by Sacha et al.(Sacha and Pluta, 2005; Sacha, 2013; Sacha et al., 2013a). Removal of this mathematical bias was achieved by the division of the SD of R-R interval (RRSD) by average R-R interval and HRV indices (LF and HF) by the average R-R interval (in seconds) squared. Corrected LF/HF ratio was calculated from corrected LF and corrected HF. After this initial mathematical correction was made, the relationships between resting HR and HRV indices (SD of R-R interval, LF variability, and HF variability) were evaluated by linear regression analysis. The resulting coefficient of determination (*r*^2^) value (i.e., *r*^2^ = 0.38) from the regression analysis was interpreted as the % change in HRV due to the prevailing HR. All reported coefficients of determination correspond to the RRSD/HR relationship. Therefore, comparison of *r*^2^-values before and after mathematical correction for prevailing HR, allowed for tracking of how prevailing HR influenced HRV during dengue viral infection. All data are presented as mean ± SD. An ANOVA with repeated measures was used for comparison between fever days.

## Results

The comparison of corrected and uncorrected HRV parameters following defervescence is presented in Table 1. By day 2, HR decreased from 98 ± 12 to 81 ± 9 beats per minute and RRI increased from 623 ± 73 to 750 ± 78 ms. Uncorrected HF and LF variability increased on Day 2 while LF/HF ratio decreased (*P* < 0.001). After correction for prevailing HR, corrected HF and LF variability were still increased, and LF/HF ratio was still decreased (*P* < 0.001) each of the 2 days following defervescence (Table 1). At defervescence (Day 0), HR accounted for ~40% (*r*^2^ = 0.38) of the variability (based on RRSD/HR relationship) before correction for HR and ~30% (*r*^2^ = 0.28) of the variability after correction for HR (normalized unit following HR correction). By Day 2 prevailing HR accounted for ~7% prior to application of HR correction and less than 1% after correction.

**Table 1:** **Heart rate variability indices with and without correction for prevailing HR**.

**Uncorrected**	**RRI (ms)**	***r*^2^ (HR to RRSD)**	**HR (b/min)**	**HF (ms^2^)**	**LF (ms^2^)**	**LF/HF**
Day 0	623 ± 74	0.38^*^	98 ± 12	194 ± 495	249 ± 381	8.2 ± 9.6
Day 1	707 ± 86	0.34	87 ± 11	405 ± 571	622 ± 751	4.1 ± 4.5
Day 2	751 ± 78^#^	0.07^#^	81 ± 9^#^	824 ± 1146^#^	1060 ± 1052^#^	2.4 ± 2.0^#^
**Corrected**		***r*^2^ (HR to RRSD)**	**HR (b/min)**	**HF**	**LF**	**LF/HF**
Day 0	–	0.28	98 ± 12	0.0003 ± 0.0008	0.0005 ± 0.0007	8.2 ± 9.6
Day 1	–	0.19	87 ± 11	0.0007 ± 0.0009	0.001 ± 0.001	4.1 ± 4.5
Day 2	–	0.004^#^	81 ± 9^#^	0.0014 ± 0.0019^#^	0.002 ± 0.001^#^	2.4 ± 2.0^#^

* *Note that HR accounted for 38% (r^2^ = 0.38) of the variability before correction for HR and 28% (r^2^ = 0.28) following HR correction*.

# *Denotes significant differences between Day 0 and Day 2. Values are presented as mean ± SD*.

To compare the absolute changes in HRV between Day 0 and Days 1 and 2 in a quantitative fashion, changes in HRV indices were calculated as percent changes (Table 2). Application of HR correction did not influence the interpretation of HF, LF, and LF/HF ratio on days 1 and 2. Specifically, uncorrected HF increased by 424 ± 120% and corrected HF increased by 377 ± 134% on day 2 (*p* = 0.45). By day 2, uncorrected LF increased by 425 ± 83% and corrected LF increased slightly less to 327 ± 96% (*P* < 0.05).

**Table 2:** **Percent changes in HRV parameters following Day 0 (defervescence) with and without HR correction**.

	**Day 1**		**Day 2**	
	**Uncorrected %**	**Corrected %**	**Uncorrected %**	**Corrected %**
HF	209 ± 43	189 ± 76	424 ± 120	377 ± 134
LF	249 ± 90	237 ± 74	425 ± 83	327 ± 96^*^
LF/HF ratio	50 ± 34	50 ± 34	29 ± 23	29 ± 23

* *Denotes significant differences in uncorrected and corrected*.

## Discussion

In general, alteration in HRV offers a clinically useful and quantifiable measure of alteration in the physiologic state of the human body. The most published HRV assessment technique for diagnosing infection is the frequency domain Fourier spectral analysis. This method; however, may be prone to erroneous results and conclusions from data due to the influence of prevailing HR. We demonstrated that the HR correction methodology used in this study was an effective way to examine HRV alterations and autonomic balance, independent of prevailing HR. The following indices of HRV were determined: (1) vagal cardiac parasympathetic activity as HF component of R-R interval variability (HF, 0.15–0.40 Hz), (2) LF component (0.04–0.15 Hz), and LF/HF ratio. The interpretation of LF/HF ration as a marker of autonomic balance has been recently questioned (Billman, 2013a,b) and has been shown to reflect major parasympathetic activity (~50%) and some sympathetic activity (Randall et al., 1991).

The present study investigated the effects of HR responses in dengue viral infected patients on HRV with and without correction for the baseline HR. Our major findings are (1) correcting HRV did not affect the direction of change in HRV parameters and (2) correcting HRV allowed for more accurate assessment of possible sympathetic (SNS) and parasympathetic nervous systems (PSNA) contributions to HRV. While we hypothesized that alterations in HRV would be minimal when corrected for prevailing HR, our data suggest that there is an autonomic regulatory basis for the HRV alterations observed with dengue viral infection independent of the influence of HR. HRV uncorrected and corrected for changes in HR revealed that cardiac parasympathetic activity likely plays major role of the HR changes following defervescence.

La-Orkhun et al. (2011) assessed HRV as an index of autonomic function in patients with DF, and found no significant changes in various time and frequency domain metrics of HRV at least 24 h after defervescence and follow-up conducted at least 14 days after defervescence. Since monitoring was performed 2 weeks after hospital discharge, it is unlikely that changes in HRV during the critical phase of illness would have been detected in their study. As such, we are the first to report HRV analysis in patients during in- hospital treatment for dengue viral infection that demonstrated significant reductions in HRV.

Several studies have examined the usefulness of HRV analysis for early diagnosis and prognosis of viral infections, particularly in neonates and infants at risk of developing septic shock (Griffin and Moorman, 2001; Griffin et al., 2004, 2005). In their studies, it was reported that abnormal HR with reduced variability and transient decelerations preceded neonatal/infant sepsis. In a study on 81 patients, Chen and Kuo showed that septic patients who subsequently developed shock had lower LF/HF ratio with respect to patients who did not develop sepsis (Chen and Kuo, 2007). In our study, the LF/HF ratio showed a progressive reduction during recovery from dengue infection (Table 1) with and without HR correction. While, it has been suggested the decreases in LF/HF ratio correspond to shift toward parasympathetic dominance (Eckberg, 1997), autonomic balance has been recently interrogated (Billman, 2013b). These adjustments in autonomic regulation are consistent with our observations in the HR response and RRI returning toward baseline values with in-hospital resuscitative treatment in DHF patients.

After correction for prevailing HR, LF variability was still significantly increased (both *P* < 0.01) on day 2 following defervescence. Furthermore, when corrected for prevailing HR, the percent change in LF variability was slightly reduced from uncorrected values of 425% to corrected values of 327%. Houle and Billman (1999) and co-workers demonstrated that the LF component of the HR power spectrum probably results from an interaction of the sympathetic and PSNA and, as such, does not precisely reveal changes in the sympathetic activity (Randall et al., 1991). These data further support our interpretation that reductions in HR following defervescence are mediated by increased cardiac parasympathetic activity and not reductions in sympathetic drive.

In conclusion, we showed that uncorrected and corrected HRV does not alter the interpretation of the potential contributions of parasympathetic and sympathetic activity in patients with dengue viral infection during their hospitalization. Additionally, HRV uncorrected and corrected for changes in HR suggest that cardiac parasympathetic activity plays an important role in HR changes following defervescence. Furthermore, the HR correction methodology employed in this study provided a unique opportunity to delineate the physiological changes in HR during treatment of dengue viral infection.

## Disclaimer

The opinions or assertions contained herein are the private views of the author and are not to be construed as official or as reflecting the views of the Department of the Army or the Department of Defense.

### Conflict of interest statement

The authors declare that the research was conducted in the absence of any commercial or financial relationships that could be construed as a potential conflict of interest.
